# Trajectories of Kidney Function in Patients with ATTRv Treated with Gene Silencers

**DOI:** 10.3390/genes13122236

**Published:** 2022-11-29

**Authors:** Marco Luigetti, Valeria Guglielmino, Angela Romano, Maria Ausilia Sciarrone, Francesca Vitali, Viola D’Ambrosio, Pietro Manuel Ferraro

**Affiliations:** 1 Fondazione Policlinico Universitario A. Gemelli IRCCS, UOC Neurologia, 00168 Roma, Italy; 2 Università Cattolica del Sacro Cuore, 00168 Roma, Italy; 3 Fondazione Policlinico Universitario A. Gemelli IRCCS, UOC Nefrologia, 00168 Roma, Italy

**Keywords:** ATTRv, amyloidosis, gene-silencing therapies, kidney involvement

## Abstract

Hereditary transthyretin amyloidosis (ATTRv; v for “variant”) is the most common form of hereditary amyloidosis, with an autosomal dominant inheritance and a variable penetrance. This disease has a significant variability in clinical presentation and multiorgan involvement. While kidney involvement in early-onset ATTRv has been reported in one-third of patients, in late-onset ATTRv it has generally been considered rare. In the present study, we describe trajectories of kidney function over time before and after treatment with gene silencing therapies in a cohort of 17 ATTRv patients with different mutations, coming from Italy (nine subjects treated with inotersen and eight patients treated with patisiran). The analysis of estimated glomerular filtration rate (eGFR) slopes revealed that the average change in eGFR was 0.01 mL/min/1.73 m^2^ per month before initiation and −0.23 mL/min/1.73 m^2^ per month during follow-up for inotersen and −0.62 mL/min/1.73 m^2^ per month before initiation and −0.20 mL/min/1.73 m^2^ per month during follow-up for patisiran. In conclusion, we did not observe any significant difference either between the two groups of treatment or within-group before and after therapy, so gene-silencing therapies may be considered safe for renal function in ATTRv and are not associated with a worsening of eGFR slope.

## 1. Introduction

Hereditary transthyretin amyloidosis (ATTRv; v for “variant”) is an autosomal dominant multisystemic disease caused by mutations in the gene encoding transthyretin (*TTR*) that results from extracellular deposition of amyloid [1]. Of the more than 120 *TTR* mutations described as a cause of ATTRv, the most frequent is the Val30Met variant [2].

Diagnosis of ATTRv amyloidosis could represent a challenge considering phenotypic variability and the multiorgan involvement. Generally, patients present with polyneuropathy, but clinicians should be able to highlight the hypothetical cardiac, ocular, and gastro-intestinal involvement [1,2,3]. However, the pattern of the clinical impairment depends on the geographic area. In endemic areas, an early onset is more frequent (third to fourth decade), and patients experience a rapid clinical course because of autonomic dysfunction and progression of the sensory–motor deficit [4]. Conversely, in non-endemic areas, the most common onset is the late one, and polyneuropathy affects predominantly the large nerve fibres. Patients progress slowly, often with cardiac involvement, and autonomic dysfunction is less frequent [5].

Kidney involvement in ATTRv usually consists of nephrotic syndrome and/or progressive renal failure. In the literature, it has been reported in about one-third of Portuguese/early-onset Val30Met patients [1,6,7]. Conversely, in late-onset cases, kidney involvement has generally been considered rare, but recent papers showed how renal function may also be impaired in 30% of late-onset ATTRv patients [8,9,10].

Current therapeutic options increase the survival of patients, reducing worsening of polyneuropathy and/or cardiomyopathy [11,12], but we do not have any therapy available for nephropathy, even if some effect of available TTR stabilizers on kidney function has been described [13].

In the present study, we describe trajectories of kidney function over time before and after treatment with gene silencing therapies in a heterogeneous Italian population of ATTRv patients.

## 2. Materials and Methods

For this study, data were collected from all patients with a diagnosis of ATTRv followed at the Neurology Unit of “Fondazione Policlinico Universitario A. Gemelli IRCCS” in Rome, Italy. The genetic mutation, age at onset and at examination, gender, and serum creatinine before and after therapy starting were collected for each patient. Estimated glomerular filtration rate (eGFR) was calculated using the race-free CKD-EPI equation [14]. In order to compute an eGFR slope, only patients with at least two measurements of serum creatinine before and after therapy starting were included. Time was modelled as a linear spline measuring months lapsed from the first measurement of serum creatinine, with one knot at the time of treatment initiation. The impact of treatment on the eGFR slope was evaluated with an interaction term between treatment groups and each spline segment.

A two-tailed *p*-value < 0.05 was considered as statistically significant. All statistical analyses were performed with Stata version 15.1 (StataCorp. 2017. Stata Statistical Software: Release 15. College Station, TX: StataCorp LLC). This study was approved by “Fondazione Policlinico Universitario A. Gemelli IRCCS”’s Ethics Committee. All patients in the study submitted informed consent. This study was carried out according to the Declaration of Helsinki.

## 3. Results

Overall, 17 patients were enrolled: nine patients were treated with inotersen, and eight patients were treated with Patisiran. Baseline characteristics of the enrolled patients are reported (Table 1 and Table 2). The majority of patients had the Val30Met variant (*n* = 6, approximately 35%) or the Phe64Leu variant (*n* = 8, 47%) (Table 1). The remaining patients harbored the Thr59Lys variant (*n* = 2, 12%), and the Ile68Leu variant (*n* = 1, 6%) (Table 1).

**Figure 1:**
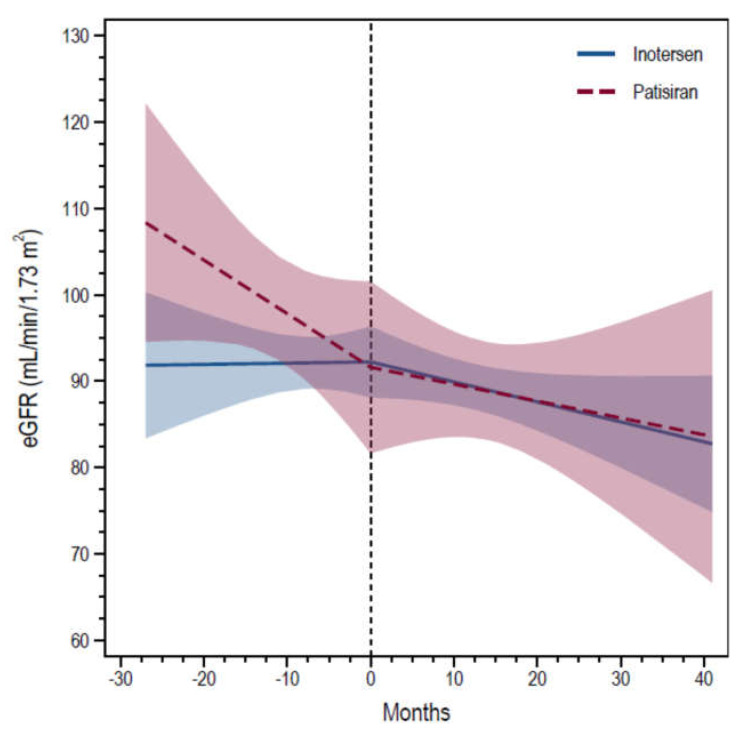
The graph reports the estimated change in eGFR over time before and after treatment initiation with inotersen (solid blue line) and patisiran (dotted red line), with shades representing 95% confidence intervals. The vertical dotted line represents treatment initiation. No significant difference either between the two groups of treatment or within-group before and after therapy (all *p*-values > 0.05) was observed.

The analysis of eGFR slopes showed that the average change in eGFR was 0.01 mL/min/1.73 m^2^ per month (95% confidence interval [CI] −0.38, 0.41) before initiation and −0.23 mL/min/1.73 m^2^ per month (95% CI −0.49, 0.03) during follow-up for inotersen, and the average change was −0.62 mL/min/1.73 m^2^ per month (95% CI −1.38, 0.14) before initiation and −0.20 mL/min/1.73 m^2^ per month (95% CI −0.79, 0.39) during follow-up for patisiran (Figure 1). Changes in eGFR were not statistically different for either inotersen (*p* = 0.94 before and *p* = 0.09 after treatment) or patisiran (*p* = 0.12 before and *p* = 0.52 after treatment). There were no differences between groups neither before (*p* = 0.15), nor after (*p* = 0.91), treatment initiation (Figure 1).

## 4. Discussion

Kidney involvement has always been considered a classical feature in ATTRv “Portuguese” phenotype but, recently, many papers also underlined its occurrence in late-onset patients [1,6,7]. The effect of new gene-silencing therapy on neuropathy progression has dramatically improved survival in ATTRv patients, and their effect on cardiological outcomes is also promising [15]. However, no therapy has been approved for renal involvement in ATTRv, and only some anecdotal reports on tafamidis are available [16].

Considering the biological effect of gene-silencers, they are able to reduce up to 80% of the circulating TTR [1]. An effect on renal amyloidosis can also be postulated, so we analysed the eGFR slope before and after gene-silencing therapy onset. Although we did not observe any significant difference, we can make some interesting considerations.

eGFR slope after starting therapy is similar for both drugs, showing a slow decline that is normal for the elderly, so an effect in preventing renal involvement due to amyloidosis can be supposed.

In the group treated with patisiran, a trend in slowing the decline can be also observed, confirming the efficacy of this drug that is the only one in which a reversal effect has been demonstrated [1].

eGFR slope in the inotersen group is stackable to the patisiran one, so the renal complications observed in the NEURO-TTR trial seem to be really rare in real-life experience [17], although a regular monitoring of renal function in these patients is always recommended.

We should also consider that our study has some limitations, such as the retrospective design, the relatively small sample size, and the lack of urinary protein excretion analysis.

In conclusion, gene-silencing therapies may be considered safe for renal function in ATTRv amyloidosis and not associated with a worsening of eGFR slope.

Future prospective collaborative studies with bigger cohorts will better clarify the effect of these new therapies on kidney function.

## Figures and Tables

**Table 1:** Demographic and clinical characteristics of the two treatment groups. Categorical variables are expressed as count and percentage (%). Numerical variables are expressed as mean ± standard deviation (SD) or median and interquartile range (IQR). N represents the number of enrolled subjects in each group.

		Inotersen	Patisiran	*p* Value
N		9	8	
Sex	Count (%)			0.93
Males		8 (89%)	7 (88%)	
Females		1 (11%)	1 (13%)	
Age at evaluation (years)	Mean (SD)	64.4 (6.7)	66.8 (5.8)	0.46
TTR variants				
Val30Met	Count (%)	4 (44%)	2 (25%)	0.25
Phe64Leu		3 (33%)	5 (63%)	
Ile68Leu		0 (0%)	1 (13%)	
Thr59Lys		2 (22%)	0 (0%)	
eGFR	Mean (SD)	93.6 (7.3)	103.6 (21.0)	0.20
Follow-up (months)	Median (IQR)	21.0 (14.0–24.0)	25.5 (13.5–29.0)	0.50

**Table 2:** Demographic and clinical data of the whole cohort of ATTRv patients. FAP, familial amyloid polyneuropathy. PND, polyneuropathy disability score. M, male. F, female.

Subject and Sex	TTR Variant	Age at Symptom Onset	Ongoing Treatment	Age at Gene-Silencing Therapy Initiation	Months since Treatment Initiation	Phenotype	FAP Stage	PND Score
M #1	Phe64Leu	64	patisiran	67	9	neuropathic	I	II
M #2	Phe64Leu	72	patisiran	75	15	mixed	I	II
M #3	Phe64Leu	69	patisiran	77	39	mixed	II	IIIa
M #4	Ile68Leu	62	patisiran	65	25	mixed	II	IIIb
M #5	Thr59Lys	46	inotersen	56	21	mixed	II	IIIa
F #6	Thr59Lys	53	inotersen	53	5	mixed	I	I
M #7	Phe64Leu	64	inotersen	69	10	mixed	II	IIIa
M #8	Phe64Leu	57	patisiran	62	12	mixed	III	IV
M #9	Val30Met	59	inotersen	64	40	mixed	II	IIIb
M #10	Phe64Leu	66	inotersen	72	22	mixed	II	IIIa
F #11	Phe64Leu	61	patisiran	68	27	neuropathic	I	II
M #12	Val30Met	53	patisiran	60	30	mixed	II	IIIb
M #13	Val30Met	66	inotersen	69	21	mixed	I	II
M #14	Val30Met	56	patisiran	67	26	mixed	I	II
M #15	Val30Met	63	inotersen	71	30	mixed	II	IIIb
M #16	Val30Met	67	inotersen	70	14	mixed	II	IIIb
M #17	Phe64Leu	57	inotersen	61	24	neuropathic	I	II

## Data Availability

The data that support the findings of this study are available from the corresponding author, upon reasonable request.
